# Effect of dietary protein on energy metabolism including protein synthesis in the spiny lobster *Sagmariasus verreauxi*

**DOI:** 10.1038/s41598-021-91304-1

**Published:** 2021-06-03

**Authors:** Shuangyao Wang, Chris G. Carter, Quinn P. Fitzgibbon, Basseer M. Codabaccus, Gregory G. Smith

**Affiliations:** Institute for Marine and Antarctic Studies (IMAS), University of Tasmania, Private Bag 49, Hobart, TAS 7001 Australia

**Keywords:** Animal physiology, Bioenergetics, Metabolism, Respiration

## Abstract

This is the first study in an aquatic ectotherm to combine a stoichiometric bioenergetic approach with an endpoint stochastic model to explore dietary macronutrient content. The combination of measuring respiratory gas (O_2_ and CO_2_) exchange, nitrogenous (ammonia and urea) excretion, specific dynamic action (SDA), metabolic energy substrate use, and whole-body protein synthesis in spiny lobster, *Sagmariasus verreauxi*, was examined in relation to dietary protein. Three isoenergetic feeds were formulated with varying crude protein: 40%, 50% and 60%, corresponding to CP_40_, CP_50_ and CP_60_ treatments, respectively. Total CO_2_ and ammonia excretion, SDA magnitude and coefficient, and protein synthesis in the CP_60_ treatment were higher compared to the CP_40_ treatment. These differences demonstrate dietary protein influences post-prandial energy metabolism. Metabolic use of each major energy substrate varied at different post-prandial times, indicating suitable amounts of high-quality protein with major non-protein energy-yielding nutrients, lipid and carbohydrate, are critical for lobsters. The average contribution of protein oxidation was lowest in the CP_50_ treatment, suggesting mechanisms underlying the most efficient retention of dietary protein and suitable dietary inclusion. This study advances understanding of how deficient and surplus dietary protein affects energy metabolism and provides approaches for fine-scale feed evaluation to support sustainable aquaculture.

## Introduction

The spiny lobster *Sagmariasus verreauxi* is the largest spiny lobster (Palinuridae) species and an important commercial seafood product in the Southern Hemisphere^1^. The recent closure of the life-cycle of *S. verreauxi* from eggs to adult in captivity has improved the pathway to sustainable aquaculture for this species^2^. Optimizing dietary protein is one key to achieving sustainable aquaculture^3–5^. A plethora of nutritional and physiological knowledge relating to dietary protein is crucial for enhancing dietary protein-sparing effects, so that the majority of assimilated protein (amino acid) can be used to improve protein synthesis retention efficiency and therefore growth, rather than for oxidation to provide metabolic energy^4–6^. Compared with many aquaculture species including other decapod crustaceans, information about the effect of dietary protein on *S. verreauxi* nutritional physiology, and other spiny lobsters in general, is limited^7–9^.

Specific dynamic action (SDA) is the increment in metabolism following feeding, representing energetic costs from ingestion, digestion, absorption and metabolic processing of energy substrates^10^. Specific dynamic action mainly represents post-absorptive metabolic costs, especially increased protein synthesis, and reflects the balance of available nutrients^4,10^. Aquafeeds with optimum digestible protein (amino acid) to energy (DP/DE) ratios and amino acid balances can reduce energy loss via SDA and improve dietary protein-sparing effects^4,11^. In contrast, imbalanced aquafeeds where the DP/DE ratio or amino acid balance is outside of the optimum range will stimulate mechanisms for regulating excess amino acids via protein synthesis or deamination and oxidation with a resultant elevation of SDA^12^. Therefore, understanding SDA and metabolic energy substrate use is essential to explore physiological mechanisms of growth, potentially helping formulate cost-effective feeds and optimizing feeding regimes^9,13,14^.

A non-destructive stoichiometric bioenergetic approach offers great potential as it can be used to examine the balance of metabolic energy substrate use in an aquatic ectotherm at any time, thus providing precise measurements on metabolic energy substrate use under different feeding conditions^6^. The use of the stoichiometric bioenergetic approach is based on the determination of respiratory quotient and nitrogen quotient, derived from the simultaneous measurement of respiratory gas (O_2_ and CO_2_) exchange and nitrogenous (ammonia and urea) excretion^15^. The stoichiometric bioenergetic approach allows repeated assessments of substrate oxidation on the same individuals^6^. However, this approach has not been widely used in aquatic ectotherms, mainly due to previous technical difficulty in accurately determining total CO_2_ (total dissolved inorganic carbon) concentrations in water^15–17^.

Protein synthesis is central to aquatic animal growth as growth occurs when whole-body protein synthesis (WBPS) exceeds protein degradation^4,18,19^. Research on protein synthesis and degradation in aquatic ectotherms has focused on fish and is less on invertebrates^10,19,20^. Investigation of WBPS in aquaculture animals provides a sensitive way to examine dietary protein (amino acid) efficiency to achieve long-term growth^18,21,22^. In aquatic ectotherms, WBPS has mainly been determined using a flooding-dose technique with an injection of a single large dose of labeled (tracer) and unlabeled (tracee) amino acid^18^. Disadvantages of this technique include it being invasive, experimental animals must be killed and measuring a suitable amino acid precursor pool for WBPS presents challenges^18^. In contrast, the development of an endpoint stochastic model enables a non-destructive measurement of WBPS in an aquatic ectotherm^23,24^. Briefly, the WBPS is measured from the cumulative excretion of stable isotope-labeled ammonia, such as ^15^N-labeled ammonia, in the excretory pool, over 24–72 h following the feeding of a single meal containing uniformly stable isotope-labeled protein, for example ^15^N-labeled *Spirulina*^18,25^. A key requirement of using the endpoint stochastic model is that the excretory pool is cleared of the stable isotope, estimated from the cumulative stable isotope excretion curve^23^. The endpoint stochastic model allows WBPS determinations in a complete daily cycle, this reduces the variation among different times of a day due to feeding and/or natural circadian rhythms, thus ensuring an integrated description of protein metabolism in aquatic ectotherms^4^. However, this approach has not yet been tested in any crustacean species.

The present study aimed to examine the effects of dietary protein on SDA, metabolic energy substrate use, and WBPS in *S. verreauxi* using a stoichiometric bioenergetic approach and an endpoint stochastic model. Intermolt lobsters were used during the whole study, including an adjunct experiment to evaluate the apparent digestibility (AD) of *Spirulina* protein, followed by an energy metabolism experiment including WBPS determination. A reference feed and a test feed were formulated and manufactured to determine the AD of *Spirulina* protein (for feed formulation, see Supplementary Table S1), and used to investigate the assimilated dose of ^15^N-labeled *Spirulina* protein to calculate WBPS^18,23^. Three isoenergetic experimental feeds containing 1% ^15^N-labeled *Spirulina* (^15^N enrichment > 98 atom% ^15^N) were formulated and manufactured with three crude protein (CP) levels: 40%, 50% and 60%, corresponding to CP_40_, CP_50_ and CP_60_ treatments, respectively (for feed formulation, see Supplementary Table S2), and used to investigate energy metabolism including protein synthesis. The oxygen consumption rate including the routine metabolic rate was determined according to methods and equipment that have been validated and used consistently in numerous experiments^26–28^. The salicylate-hypochlorite method, the modified diacetyl monoxime method, and the infrared detection method were used to determine the total ammonia-N excretion rate, urea-N excretion rate, and total dissolved inorganic carbon excretion rate, respectively, and detailed in Wang et al*.*^9^. The results from this study improve the understanding of how dietary protein affects energy metabolism including protein synthesis in aquatic ectotherms, which provides a physiological basis of growth and is essential to optimize feeds and feeding regimes in aquaculture.

## Results

### Spirulina and lobster chemical composition and apparent digestibility of Spirulina protein

The dry matter, crude protein, total lipid, carbohydrate, ash and gross energy in *Spirulina* were 97.0%, 70.7%, 7.0%, 13.8%, 8.5% and 21.1 kJ g^−1^ dry matter (DM), respectively; and 28.4%, 58.4%, 10.4%, 7.9%, 23.3% and 17.5 kJ g^−1^ DM, respectively, in whole-body *S. verreauxi*. The apparent digestibility (AD) of *Spirulina* protein was 52.9%.

### Parameters in SDA

**Figure 1:**
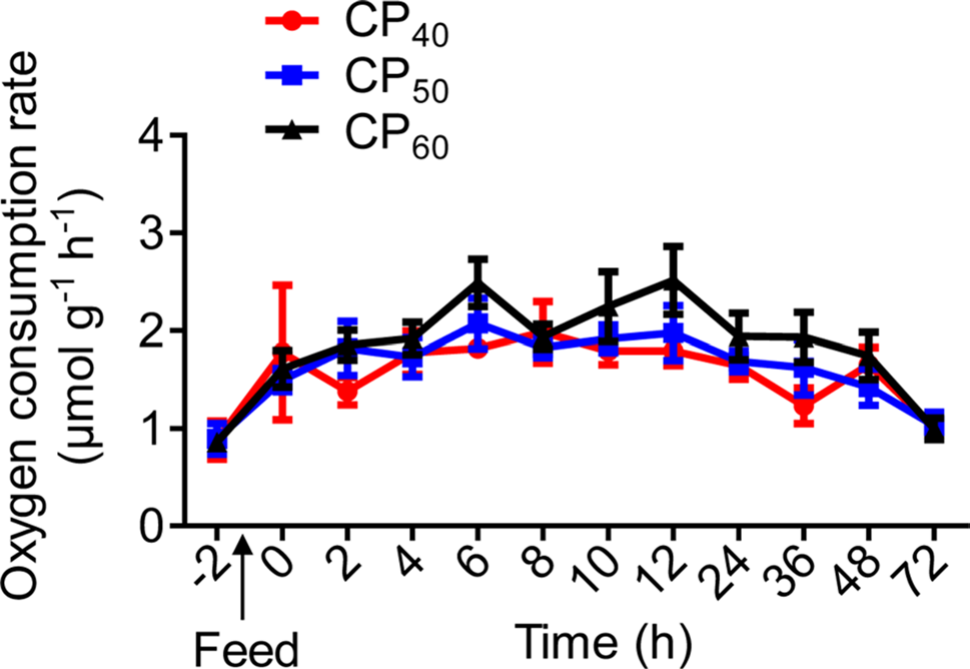
Oxygen consumption rate (*M*O_2_, μmol g^−1^ h^−1^) in juvenile *Sagmariasus verreauxi*. Lobsters were reared at 21 °C and fed ^15^N-labeled feeds at 1.5% body weight at three crude protein levels: 40%, 50% and 60%, corresponding to CP_40_, CP_50_ and CP_60_ treatments, respectively. The *M*O_2_ at − 2 h indicates the routine metabolic rate (RMR). The first post-prandial *M*O_2_ was recorded at 0 h. All data represent mean ± standard error (SE) of 6 individuals.

There were no differences in the routine metabolic rate (RMR), peak SDA (SDA_peak_), time to SDA_peak_, or SDA duration among treatments (Table 1, Fig. 1). The energetic cost of SDA (E_SDA_) evaluated by different approaches was similar in each treatment (Table 1). The SDA magnitude, E_SDA_ and SDA coefficient (C_SDA_) in the CP_60_ treatment were higher (*P* < 0.05) compared to the CP_40_ treatment, and there were no differences between CP_40_ and CP_50_ or between CP_50_ and CP_60_ treatments (Table 1).

**Table 1:** Characteristics of the specific dynamic action (SDA) response in juvenile *Sagmariasus verreauxi*.

Parameters	CP_40_	CP_50_	CP_60_
RMR (μmol g^−1^ h^−1^)	0.88 ± 0.20	0.89 ± 0.16	0.86 ± 0.10
SDA_peak_ (μmol g^−1^ h^−1^)	2.63 ± 0.52	2.24 ± 0.23	2.91 ± 0.22
Time to SDA_peak_ (h)	6 ± 1.86	10.33 ± 2.99	11.67 ± 2.70
SDA duration (h)	64 ± 8	72 ± 0	66 ± 6
SDA magnitude (μmol g^−1^)	12.82 ± 1.05^a^	17.32 ± 1.79^ab^	18.60 ± 2.00^b^
E_SDA_ (J g^−1^) calculated by the empirical Q_ox_ approach	5.68 ± 0.46^a^	7.67 ± 0.79^ab^	8.24 ± 0.89^b^
E_SDA_ (J g^−1^) calculated by the composite Q_ox_ approach	5.74 ± 0.47^a^	7.65 ± 0.79^ab^	8.10 ± 0.87^b^
E_SDA_ (J g^−1^) calculated by the stoichiometric approach	5.81 ± 0.40^a^	7.54 ± 0.73^ab^	8.53 ± 0.87^b^
C_SDA_ (%)	1.80 ± 0.15^a^	2.45 ± 0.25^ab^	2.59 ± 0.28^b^

Lobsters were reared at 21 °C and fed ^15^N-labeled feeds at 1.5% body weight at three crude protein levels: 40%, 50% and 60%, corresponding to CP_40_, CP_50_ and CP_60_ treatments, respectively.

RMR, routine metabolic rate; SDA_peak_, peak SDA; E_SDA_, SDA magnitude converted to energy; Q_ox_, oxycalorific coefficient; C_SDA_, SDA coefficient. All data represent mean ± standard error (SE) of 5 to 6 individuals. Different superscripts (a, b) in each row indicate significant differences among treatments (One-way ANOVA, *P* < 0.05).

### Nitrogenous excretion and nitrogen quotient

**Figure 2:**
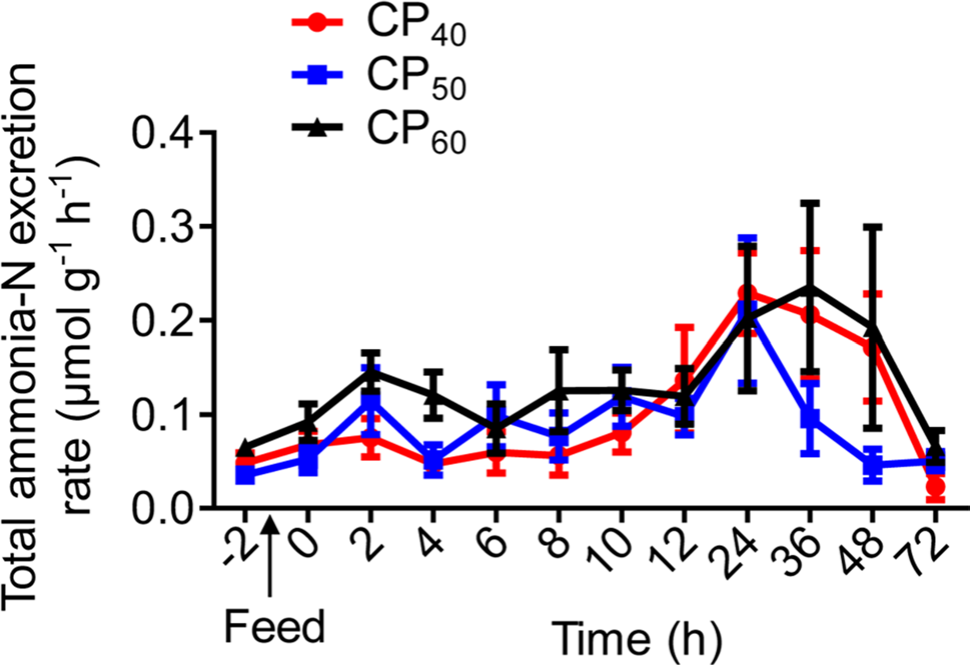
Total ammonia-N excretion rate (*M*TAN, μmol g^−1^ h^−1^) in juvenile *Sagmariasus verreauxi*. Lobsters were reared at 21 °C and fed ^15^N-labeled feeds at 1.5% body weight at three crude protein levels: 40%, 50% and 60%, corresponding to CP_40_, CP_50_ and CP_60_ treatments, respectively. The *M*TAN at − 2 h indicates the routine *M*TAN. The first post-prandial *M*TAN was recorded at 0 h. All data represent mean ± standard error (SE) of 6 individuals.

**Figure 3:**
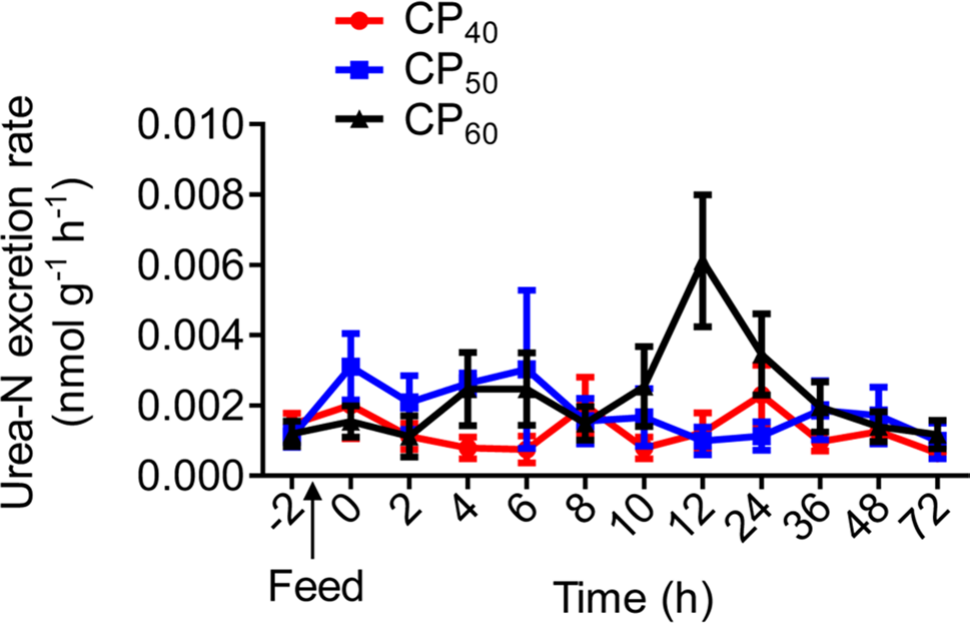
Urea-N excretion rate (*M*urea-N, nmol g^−1^ h^−1^) in juvenile *Sagmariasus verreauxi*. Lobsters were reared at 21 °C and fed ^15^N-labeled feeds at 1.5% body weight at three crude protein levels: 40%, 50% and 60%, corresponding to CP_40_, CP_50_ and CP_60_ treatments, respectively. The *M*urea-N at − 2 h indicates the routine *M*urea-N. The first post-prandial *M*urea-N was recorded at 0 h. All data represent mean ± standard error (SE) of 6 individuals.

There were no differences in the routine total ammonia-N excretion rate (*M*TAN), peak *M*TAN (*M*TAN_peak_), time to *M*TAN_peak_, or *M*TAN duration among treatments (Table 2, Fig. 2). The *M*TAN magnitude in the CP_60_ treatment during SDA was higher (*P* < 0.05) compared to the CP_40_ treatment, and there were no differences between CP_40_ and CP_50_ or between CP_50_ and CP_60_ treatments (Table 2). There were no differences in the routine urea-N excretion rate (*M*urea-N), peak *M*urea-N (*M*urea-N_peak_), time to *M*urea-N_peak_, or *M*urea-N duration among treatments (Table 2, Fig. 3). The *M*urea-N magnitude in CP_50_ and CP_60_ treatments during SDA was higher (*P* < 0.05) compared to the CP_40_ treatment (Table 2).

**Table 2:** Characteristics of total ammonia-N (*M*TAN) and urea-N (*M*urea-N) excretion in juvenile *Sagmariasus verreauxi*.

Parameters	CP_40_	CP_50_	CP_60_
Routine *M*TAN (μmol g^−1^ h^−1^)	0.048 ± 0.01	0.035 ± 0.01	0.065 ± 0.01
*M*TAN_peak_ (μmol g^−1^ h^−1^)	0.31 ± 0.05	0.28 ± 0.06	0.35 ± 0.08
Time to *M*TAN_peak_ (h)	28 ± 4	19.33 ± 5.23	25 ± 6.96
*M*TAN duration (h)	62 ± 6.51	46 ± 5.73	54 ± 6
*M*TAN magnitude (μmol g^−1^)	1.62 ± 0.14^a^	1.88 ± 0.15^ab^	2.37 ± 0.21^b^
Routine *M*urea-N (nmol g^−1^ h^−1^)	0.0015 ± 0.00032	0.0011 ± 0.00030	0.0012 ± 0.00036
*M*urea-N_peak_ (nmol g^−1^ h^−1^)	0.0042 ± 0.00064	0.0060 ± 0.0017	0.0077 ± 0.0014
Time to *M*urea-N_peak_ (h)	12.33 ± 4.01	7.67 ± 5.74	13 ± 2.41
*M*urea-N duration (h)	22 ± 4.82	24 ± 6.20	34 ± 3.69
*M*urea-N magnitude (nmol g^−1^)	0.0075 ± 0.00042^a^	0.013 ± 0.0025^b^	0.022 ± 0.0030^b^

Lobsters were reared at 21 °C and fed ^15^N-labeled feeds at 1.5% body weight at three crude protein levels: 40%, 50% and 60%, corresponding to CP_40_, CP_50_ and CP_60_ treatments, respectively.

*M*TAN_peak_, peak *M*TAN; *M*urea-N_peak_, peak *M*urea-N. All data represent mean ± standard error (SE) of 6 individuals. Different superscripts (a, b) in each row indicate significant differences among treatments (One-way ANOVA, *P* < 0.05).

**Figure 4:**
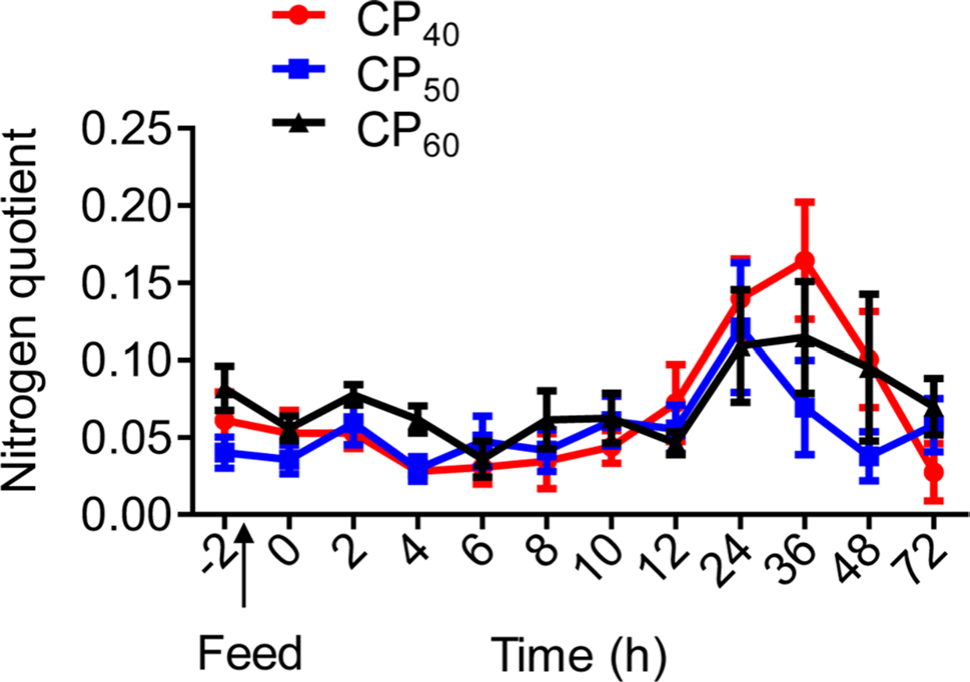
Nitrogen quotient (NQ) in juvenile *Sagmariasus verreauxi*. Lobsters were reared at 21 °C and fed ^15^N-labeled feeds at 1.5% body weight at three crude protein levels: 40%, 50% and 60%, corresponding to CP_40_, CP_50_ and CP_60_ treatments, respectively. The NQ at − 2 h indicates the routine NQ. The first post-prandial NQ was recorded at 0 h. All data represent mean ± standard error (SE) of 6 individuals.

In the CP_60_ treatment, the routine nitrogen quotient (NQ) was higher (Paired t-tests, *P* = 0.024) than that at 6 h post-feeding, and there were no differences between the routine and other post-prandial NQ (Fig. 4). There were no differences between the routine and post-prandial NQ in the CP_50_ treatment at different time periods (Fig. 4). In the CP_40_ treatment, the routine NQ was higher (Paired t-tests, *P* = 0.032) than that at 6 h post-feeding, and lower than that at 24 and 36 h post-feeding (Paired t-tests, *P* = 0.012 and 0.047, respectively) (Fig. 4).

### Total dissolved inorganic carbon excretion and respiratory quotient

**Figure 5:**
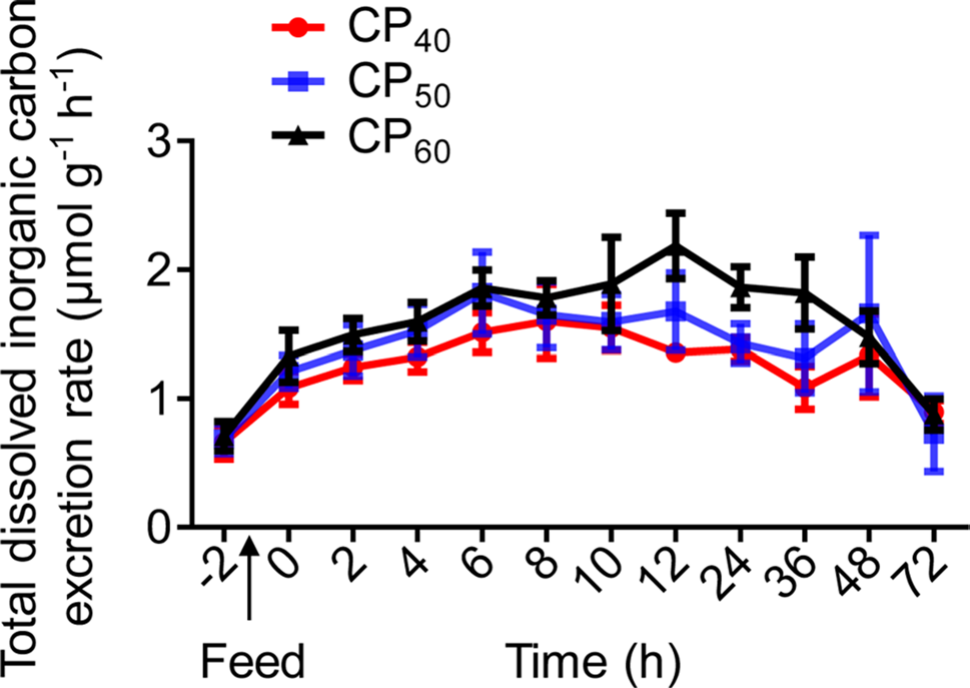
Total dissolved inorganic carbon excretion rate (*M*DIC, μmol g^−1^ h^−1^) in juvenile *Sagmariasus verreauxi*. Lobsters were reared at 21 °C and fed ^15^N-labeled feeds at 1.5% body weight at three crude protein levels: 40%, 50% and 60%, corresponding to CP_40_, CP_50_ and CP_60_ treatments, respectively. The *M*DIC at − 2 h indicates the routine *M*DIC. The first post-prandial *M*DIC was recorded at 0 h. All data represent mean ± standard error (SE) of 5 to 6 individuals.

**Figure 6:**
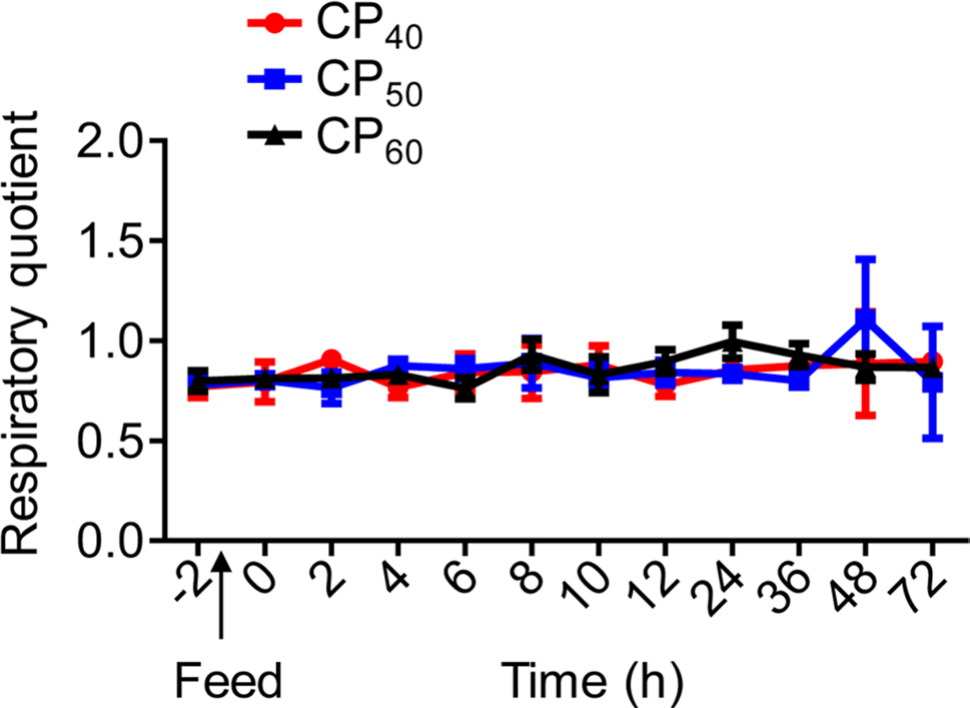
Respiratory quotient (RQ) in juvenile *Sagmariasus verreauxi*. Lobsters were reared at 21 °C and fed ^15^N-labeled feeds at 1.5% body weight at three crude protein levels: 40%, 50% and 60%, corresponding to CP_40_, CP_50_ and CP_60_ treatments, respectively. The RQ at − 2 h indicates the routine RQ. The first post-prandial RQ was recorded at 0 h. All data represent mean ± standard error (SE) of 5 to 6 individuals.

There were no differences in the routine total dissolved inorganic carbon excretion rate (*M*DIC), peak *M*DIC (*M*DIC_peak_), time to *M*DIC_peak_, or *M*DIC duration among treatments (Table 3, Fig. 5). The *M*DIC magnitude in the CP_60_ treatment during SDA was higher (*P* < 0.05) compared to the CP_40_ treatment, and there were no differences between CP_40_ and CP_50_ or between CP_50_ and CP_60_ treatments (Table 3). There were no differences between routine and post-prandial respiratory quotient (RQ) at different time periods in all treatments (Fig. 6).

**Table 3:** Characteristics of total dissolved inorganic carbon excretion (*M*DIC) in juvenile *Sagmariasus verreauxi*.

Parameters	CP_40_	CP_50_	CP_60_
Routine *M*DIC (μmol g^−1^ h^−1^)	0.66 ± 0.13	0.69 ± 0.11	0.71 ± 0.12
*M*DIC_peak_ (μmol g^−1^ h^−1^)	2.15 ± 0.20	2.61 ± 0.43	2.50 ± 0.23
Time to *M*DIC_peak_ (h)	17.67 ± 6.60	14 ± 6.85	18.33 ± 5.64
*M*DIC duration (h)	50 ± 7.85	46 ± 6.51	62 ± 6.51
*M*DIC magnitude (μmol g^−1^)	13.25 ± 1.25^a^	14.84 ± 2.66^ab^	20.12 ± 2.31^b^

Lobsters were reared at 21 °C and fed ^15^N-labeled feeds at 1.5% body weight at three crude protein levels: 40%, 50% and 60%, corresponding to CP_40_, CP_50_ and CP_60_ treatments, respectively.

*M*DIC_peak_, peak *M*DIC. All data represent mean ± standard error (SE) of 5 to 6 individuals. Different superscripts (a, b) in each row indicate significant differences among treatments (One-way ANOVA, *P* < 0.05).

### Instantaneous metabolic energy substrate use

**Figure 7:**
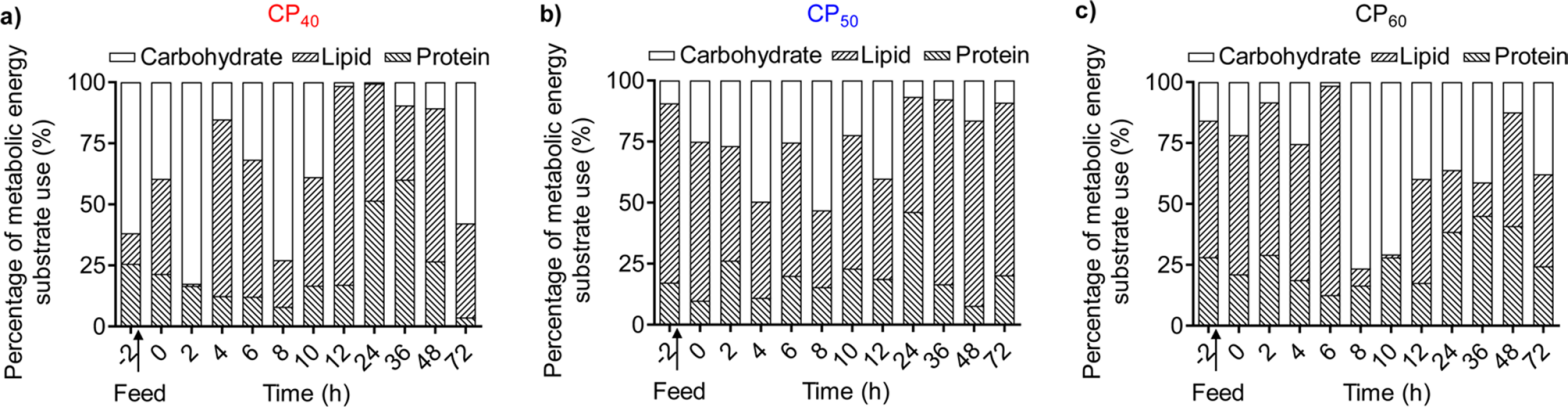
Instantaneous metabolic energy substrate use (%) in juvenile *Sagmariasus verreauxi*. Lobsters were reared at 21 °C and fed ^15^N-labeled feeds at 1.5% body weight at three crude protein levels: 40%, 50% and 60%, corresponding to CP_40_, CP_50_ and CP_60_ treatments, respectively. The percentage at − 2 h indicates the metabolic energy substrate use during routine metabolism. The first post-prandial metabolic energy substrate use was recorded at 0 h. All data represent mean values of 4 to 6 individuals.

In the CP_40_ treatment, carbohydrate was the predominant metabolic energy substrate in 3-day fasted lobsters, followed by protein (amino acid), and lipid oxidation played a minor role (Fig. 7a). During the 72 h post-feeding, the average fractional contributions of protein (amino acid), lipid and carbohydrate to metabolic use were 22.4%, 44.8% and 32.8%, respectively. Protein (amino acid) contribution decreased in the first 8 h after feeding, then increased from 8 to 36 h post-feeding, and thereafter decreased in 36–72 h post-feeding. Lipid contribution fluctuated during the SDA process. The lowest lipid contribution (0.9%) was at 2 h post-feeding and the highest (81.4%) at 12 h. Carbohydrate contribution fluctuated in 0–8 h post-feeding, then decreased from 8 to 24 h post-feeding, and thereafter increased in 24–72 h post-feeding (Fig. 7a).

In the CP_50_ treatment, lipid was preferentially oxidized for energy production in 3-day fasted lobsters, followed by protein (amino acid), and carbohydrate oxidation played a minor role (Fig. 7b). During the 72 h post-feeding, the average fractional contributions of protein (amino acid), lipid and carbohydrate to metabolic use were 19.6%, 54.8% and 25.6%, respectively. In the first 12 h after feeding, protein (amino acid) oxidation remained stable, lipid oxidation decreased and carbohydrate oxidation increased. From 12 to 24 h post-feeding, protein (amino acid) and lipid oxidation increased and carbohydrate oxidation decreased. From 24 to 72 h post-feeding, protein (amino acid) oxidation decreased, lipid oxidation increased and carbohydrate oxidation remained low (Fig. 7b).

In the CP_60_ treatment, lipid was the main metabolic energy substrate in 3-day fasted lobsters, followed by protein (amino acid), and carbohydrate oxidation played a minor role (Fig. 7c). During the 72 h post-feeding, the average fractional contributions of protein (amino acid), lipid and carbohydrate to metabolic use were 26.7%, 39.6% and 33.7%, respectively. In the first 36 h after feeding, although there was some fluctuation among time-blocks there was a trend for increased protein (amino acid) and carbohydrate oxidation and decreased lipid oxidation. From 36 to 48 h post-feeding, protein (amino acid) and carbohydrate oxidation decreased and lipid oxidation increased. From 48 to 72 h post-feeding, protein (amino acid) and lipid oxidation decreased and carbohydrate oxidation increased (Fig. 7c).

### Whole-body protein synthesis

**Figure 8:**
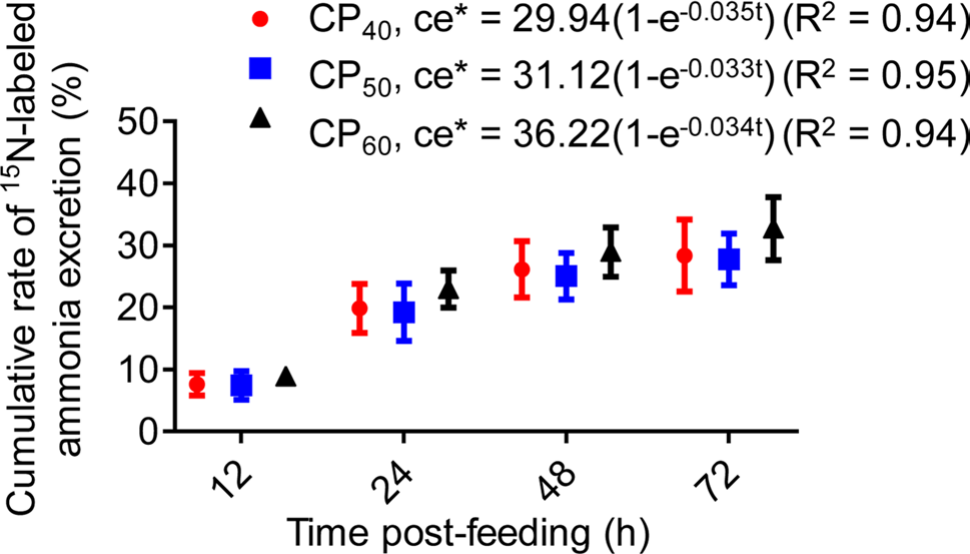
Cumulative rate of ^15^N-labeled ammonia excretion (ce*, %, expressed as the percentage of ^15^N in the assimilated feed) in juvenile *Sagmariasus verreauxi*. Lobsters were reared at 21 °C and fed ^15^N-labeled feeds at 1.5% body weight at three crude protein levels: 40%, 50% and 60%, corresponding to CP_40_, CP_50_ and CP_60_ treatments, respectively. All data represent mean ± standard error (SE) of 6 individuals.

**Figure 9:**
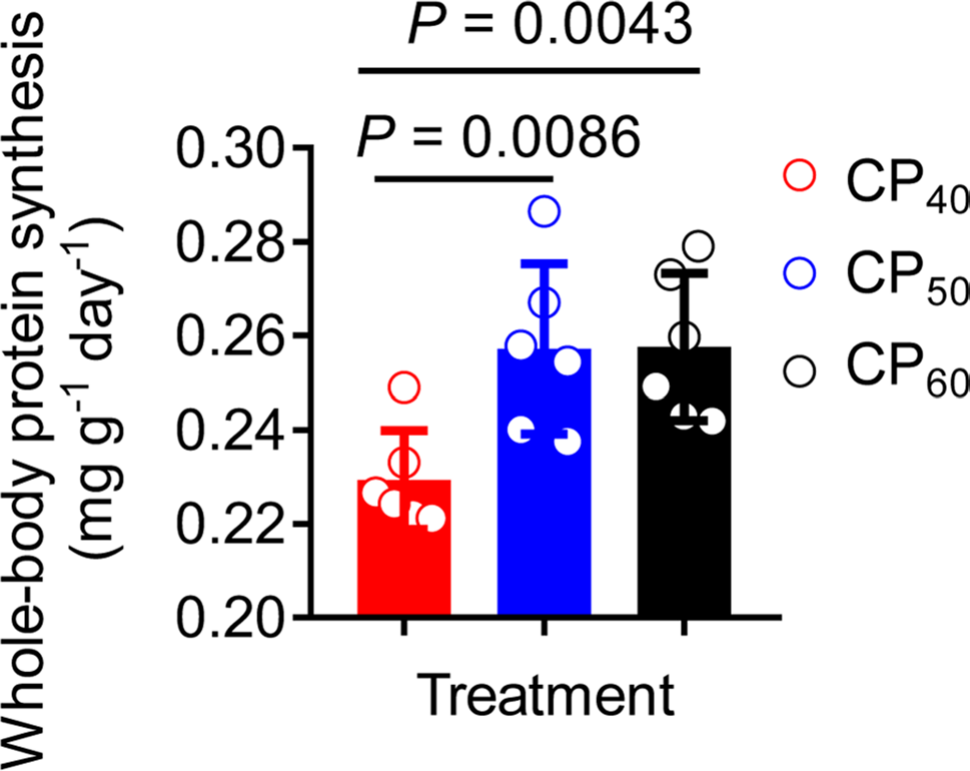
Whole-body protein synthesis (WBPS, mg g^−1^ day^−1^) in juvenile *Sagmariasus verreauxi* estimated over 48 h post-feeding. Lobsters were reared at 21 °C and fed ^15^N-labeled feeds at 1.5% body weight at three crude protein levels: 40%, 50% and 60%, corresponding to CP_40_, CP_50_ and CP_60_ treatments, respectively. All data represent mean ± standard error (SE) of 6 individuals.

The cumulative rate of ^15^N-labeled ammonia excretion after feeding (ce*) in all treatments had significant negative exponential regressions over time (t) (Fig. 8). In all treatments, ce* was different among 12–72 h post-feeding (one-way repeated-measures ANOVA, *P* < 0.05) and similar between 48 and 72 h post-feeding (Tukey HSD test, *P* > 0.05). Therefore, the whole-body protein synthesis (WBPS) was estimated using data collected over 48 h post-feeding, during which the WBPS was 0.23 ± 0.004, 0.26 ± 0.007, and 0.26 ± 0.006 mg g^−1^ day^−1^, respectively, in CP_40_, CP_50_, and CP_60_ treatments (Fig. 9). The fractional protein synthesis rate (k_s_) was also estimated using data collected over 48 h post-feeding, during which the k_s_ was 0.17 ± 0.003, 0.19 ± 0.005, and 0.19 ± 0.004% day^−1^, respectively, in CP_40_, CP_50_, and CP_60_ treatments. In CP_60_ and CP_50_ treatments, the WBPS and k_s_ estimated over 48 h post-feeding were higher (*P* < 0.05) compared to the CP_40_ treatment.

## Discussion

This study for the first time in an aquatic ectotherm successfully combined a stoichiometric bioenergetic approach used to evaluate metabolic substrate use and specific dynamic action (SDA) with an endpoint stochastic model used to assess whole-body protein synthesis (WBPS). Protein synthesis and SDA magnitude increased with dietary protein. The metabolic use of each major energy substrate varied at different time periods during the SDA process, and the average contribution of protein (amino acid) oxidation was lowest in the treatment with 50% dietary protein. The approaches and findings from this comprehensive study extend the understanding of detailed nutritional physiology and bioenergetics in crustaceans to support the continuous development and refinement of sustainable and cost-effective aquafeeds^12,14,29^.

The energetic cost of SDA (E_SDA_) estimated by three different approaches (two simplified traditional approaches and one stoichiometric bioenergetic approach) within every treatment was similar and consistent with other studies in aquatic ectotherms^9,30,31^. These findings suggest that traditional approaches would provide adequate information on E_SDA_; however, more studies on a variety of aquatic ectotherms are required to further identify whether the estimation is comparable among approaches^17^. As with other decapods fed isoenergetic feeds^32–34^, lobster SDA magnitude increased with dietary protein. A growing body of evidence suggests that the major part of SDA in aquatic ectotherms is attributable to WBPS^26,35,36^. Hence, the increased SDA magnitude with increased dietary protein indicates increased WBPS.

The SDA coefficient (C_SDA_) is the most informative parameter in SDA, reflecting the metabolic use of digestible energy and dietary protein^36–38^. For example, the C_SDA_ in carnivorous southern catfish, *Silurus meridionalis*, increased with increasing dietary soybean protein while the growth performance decreased, suggesting that imbalanced amino acids due to the replacement of fish meal by soybean meal may result in more energy used for metabolism^39^. A model has been presented to explain how different levels of dietary protein and essential amino acid supply, ranging from deficiency to excess, influence the stimulation of protein synthesis, amino acid oxidation and energy expenditure, and ultimately protein retention efficiency^4^. Therefore, the combination of C_SDA_ with nitrogenous excretion further improves the understanding of dietary protein use^13^. For example, increased C_SDA_ and nitrogenous excretion indicates increased protein metabolism and decreased efficiency of transformation of ingested nutrients to growth^32,34,40^. In contrast, optimal dietary protein may result in low C_SDA_ and nitrogenous excretion^13^. For example, the C_SDA_ and nitrogenous excretion in the postlarvae of the shrimp *Penaeus notiatis* increased when dietary protein increased from 40 to 65%, indicating 40% dietary protein may meet the postlarval requirement^32^. The C_SDA_ and nitrogenous excretion in juvenile *P. setiferus* were lowest when dietary protein was 30% and increased when dietary protein was below or above 30%, suggesting 30% dietary protein may spare dietary protein more efficiently^34^. Similarly, the C_SDA_ and nitrogenous excretion in the subadult white shrimp, *Litopenaeus vannamei*, increased when dietary protein increased from 20 to 50%, indicating 20% dietary protein may spare dietary protein more efficiently^40^. As with other decapods fed isoenergetic feeds^32,40^, the C_SDA_ and post-prandial ammonia excretion in the present study increased with dietary protein. In contrast, compared with previous research in the same or related spiny lobster species, *Jasus edwardsii*, fed squid rich in protein^26,38,41^, the C_SDA_ in the present study was low. Since C_SDA_ is independent of body weight^37,42^, the low C_SDA_ in the present study indicates efficient use of the provided formulated feeds in comparison to squid feeds^34,39,43^.

In the present study, ammonia excretion was similar to other decapod crustaceans^44,45^. Ammonia was the major nitrogenous end-product and urea contribution was negligible and there was a large 4–8 fold increase following feeding^38,46,47^. The magnitude of nitrogenous and CO_2_ excretion during SDA in the present study increased with dietary protein, indicating high-protein feeds provided substrates for both protein synthesis and energy to fuel protein synthesis^4,13,45^.

The investigation of instantaneous metabolic energy substrate use helps understand how aquatic ectotherms oxidize energy substrates to provide energy at different nutritional status and evaluate the dietary protein-sparing effect^9,15,48^. The major energy substrates oxidized in 3-day fasted lobsters was lipid in CP_60_ and CP_50_ treatments and was carbohydrate in the CP_40_ treatment, differing from a previous study in *S. verreauxi* where protein (amino acid) oxidation predominated^9^. This discrepancy could be due to the variation in previous nutritional history^12,13,49^. The previous study fed *S. verreauxi* high-protein natural feed before experimentation^9^ and the present study fed formulated feeds at different dietary protein before short-term fasting. In all treatments, ingested protein (amino acid), lipid and carbohydrate were all oxidized at different proportions at different time periods over 72 h post-feeding, indicating that suitable amounts of high-quality protein with both major non-protein energy-yielding nutrients, lipid and carbohydrate, are critical for the formulation of balanced and cost-effective feeds to spiny lobsters^9,50^. The average fractional contribution of protein (amino acid) oxidation to total energy production during the SDA process was lowest in the CP_50_ treatment and highest in the CP_60_ treatment, suggesting that the feed containing 50% crude protein, 15% total lipid and 25% carbohydrate may spare dietary protein more efficiently. There is little information on protein requirements in spiny lobsters^7,8,50^ and the optimum dietary protein in juvenile *S. verreauxi* is still unknown. Long-term growth experiments in *J. edwardsii* showed that a feed containing 32% crude protein, 14% total lipid and 27% carbohydrate was suitable for juvenile growth^51^. In contrast, another study showed that a feed containing 40% crude protein and 9% total lipid (carbohydrate content was not given) was more suitable for *J. edwardsii* growth^8^. The higher protein requirement recorded in the present study compared with *J. edwardsii*^8,51^ could be due to different dietary protein sources. The present study used casein and mussel at a ratio of 2:1, while studies in *J. edwardsii* used fish meal as the main protein sources^8,51^, which may result in different dietary amino acid profiles^52^. The total lipid to carbohydrate ratio in the most efficient feed in the present study was 1:2, in accordance with^51^. Hence, the stoichiometric estimation of metabolic energy substrate use suggests that the stoichiometric bioenergetic approach may provide a promising and efficient alternative to evaluate nutrient compositions.

The investigation of WBPS is the key to understanding daily protein-nitrogen flux in aquatic ectotherms, critical for optimizing dietary protein to formulate cost-effective aquafeeds^19,22,53^. The present study, for the first time, used an endpoint stochastic model to measure WBPS in crustaceans. To determine *S. verreauxi* WBPS, the apparent digestibility of *Spirulina* protein was evaluated. As with the prawn *Macrobrachium tenellum*^54^, the apparent digestibility of *Spirulina* protein for *S. verreauxi* was 53%. Using the endpoint stochastic model to validly determine WBPS requires ^15^N-labeled nitrogen appearing in a single major nitrogenous end-product^19,23,24^. Most previous studies in aquatic ectotherms used ^15^N-labeled ammonia to calculate WBPS as urea excretion was low^19,24,55^. In the present study, urea excretion was negligible. Therefore, the use of ^15^N-labeled ammonia to calculate WBPS in *S. verreauxi* is acceptable. The validation data in the present study showed that as with other aquatic ectotherms^19,23,56^, the cumulative ^15^N-labeled ammonia excretion rates in *S. verreauxi* were constant at 48 h post-feeding.

There has been little work on post-prandial WBPS in crustaceans^36,49,57^. In the crab *Carcinus maenas* (15–18 °C, 54 g) fed at 3% BW, the WBPS ranged from 0.8 to 1.6 mg g^−1^ day^−1^ at different post-prandial times^49^. In the isopod *Glyptonotus antarcticus* (0 °C, 33 g) fed at 5% BW, the WBPS was 0.13 mg g^−1^ day^−1^ at the SDA_peak_^36^. In the isopod *Saduria entomon* (1 g) fed at 5% BW at 4 °C and 13 °C, the WBPS at the SDA_peak_ was 0.6 and 1.0 mg g^−1^ day^−1^, respectively^57^. The large WBPS variation can be partly explained by the combination of factors in each experiment including environmental temperature, body weight, and feeding regimes^53,57,58^. Moreover, different species may also result in various WBPS^12^. For example, the routine WBPS in *S. entomon*^57^ was three times higher than that in *G. antarticus* at 4 °C^36^. The whole-body protein content estimated in the present study was in line with *S. verreauxi* puerulus^59^ and juvenile *J. edwardsii*^8,41,51^. The change of the whole-body fractional protein synthesis rate (k_s_) was comparable to WBPS change among treatments.

This study was the first to examine the relationship between WBPS and dietary protein in a crustacean species. As with other aquatic ectotherms^33,49,60^, *S. verreauxi* WBPS increased with dietary protein. In combination with the findings in the SDA magnitude and metabolic energy substrate use, the present study suggests that a formulated feed containing 50% crude protein can satisfy *S. verreauxi* protein requirement.

## Conclusion

This study comprehensively examined the effects of dietary protein on all major components of whole-animal metabolism: respiratory gas exchange, nitrogenous excretion, SDA, metabolic energy substrate use, and whole-body protein synthesis in *S. verreauxi*, with the use of a stoichiometric bioenergetic approach and an endpoint stochastic model. Dietary protein had significant influence on routine and post-prandial nutritional physiology and bioenergetics. A balanced feed containing suitable protein (amino acid), lipid and carbohydrate is essential to spare dietary protein for spiny lobsters, and a feed containing 50% crude protein appears to best satisfy *S. verreauxi* protein requirement. The comprehensive results advance the knowledge of how deficient and surplus dietary protein affects energy metabolism and provide approaches for fine-scale feed evaluation, critical for achieving more sustainable aquaculture through refinement of feeds with an emphasis on maximizing the use of dietary protein for growth. In future, using different indirect calorimetric approaches to estimate the energetic cost of SDA should be expanded to assess the availability of simplified traditional approaches.

## Methods

### Lobster husbandry

*Sagmariasus verreauxi* were hatchery reared from eggs at the Institute for Marine and Antarctic Studies (IMAS), Hobart, Australia^61^. Thirty juveniles [body weight (BW), 993 ± 23 g (mean ± standard error (SE)), range 750–1150 g] were evenly and randomly divided into three identical 200 L rectangular cages (A, B, C) made of oyster mesh (5 mm mesh size), which were floating in a 4000-L fiberglass tank supplied with flow-through filtered seawater and aerated by an air-stone connected to a central air supply. The male–female sex ratio was 1:1 in each cage. The tank was covered with black plastic to decrease lobster visual disturbance. Seawater quality was maintained at temperature 21 ± 0.2 °C, salinity 35 ± 0.1 ppt, pH 8.1 ± 0.1, dissolved oxygen 100 ± 10.0% saturation. To avoid interference from circadian rhythms, lobsters were acclimated to constant dim light for 4 weeks before experimentation^26^. During acclimation, lobsters were fed ad libitum fresh blue mussels *Mytilus galloprovincialis* twice a week at 08:00. Animal ethics were not required in this study, but utmost care was still provided to the lobsters.

### Feed formulation and manufacturing-Spirulina protein apparent digestibility experiment

All chemicals used in this study were purchased from Sigma-Aldrich (Castle Hill, NSW, Australia) unless noted otherwise. Yttrium oxide (Y_2_O_3_) (1 g kg^−1^) as an inert digestibility marker was added into the feeds at the start of feed preparation^62^. Briefly, all finely ground dry ingredients were thoroughly mixed with a mixer (Premier Chef KMC510, Kenwood, London, UK) for 20 min, after which krill oil, lecithin and de-ionized water (40 °C) were added to form a soft dough. The dough was then cold extruded through a 10 mm die using a Dolly Pasta Machine (LaMonferrina, Italy). Thereafter, the pellets were cut to 5 cm in length appropriate for the experimental lobsters and kept in air-tight containers at 4 °C overnight to complete the enzymatic binding process, then placed into a fan-ventilated oven (Steridium, Australia) at 40 °C for an hour to attain a moisture content less than 30%^7,8^ and cooled for 15 min. The cooled pellets (feeds) were packed in sealed bags and stored at − 20 °C until used.

### Feed formulation and manufacturing-energy metabolism including protein synthesis experiment

Apart from the three ^15^N-labeled experimental feeds as described above, another three feeds used in a 1-week acclimation phase before the experiment were also formulated. The chemical composition in the feeds used in acclimation was the same as that in the three experimental feeds, except that 1% normal *Spirulina* (^14^N-labeled), instead of ^15^N-labeled *Spirulina* was used. All feeds were manufactured using the same procedure and dimensions as the adjunct experiment measuring the AD of *Spirulina* protein.

### Apparent digestibility of Spirulina protein

After the 4-week acclimation, two lobsters from each cage were randomly chosen, weighed and transferred individually by a net into 30 L identical blue polyethylene tanks (width, 30 cm; length, 40 cm; height, 25 cm; N = 6) containing 16 L of running (flow rate: 80 L min^−1^) and aerated seawater. The six lobsters (BW, 1017 ± 60 g, range 750–1150 g, male : female sex ratio = 1:1) were reared under the same conditions as acclimation for 3 weeks. Artificial shelters made of oyster mesh were placed on the bottom to provide the lobsters with substrates to hold to minimize stress. Three of the lobsters were randomly chosen and fed the reference feed twice a day by hand at 1% BW at 08:00 and 2% BW at 18:00 for a week, and the remaining three lobsters were fed the test feed using the same method. Lobsters were allowed 30 min to consume the feeds followed by siphoning of feed residues. Faeces were siphon-collected onto a 500-μm mesh screen hourly from 09:00 to 21:00 to determine the peak time point of egestion. Preliminary observations showed that lobsters fed the reference and test feeds egested the largest amount of faeces at 4–7 h and 5–8 h post-feeding, respectively. Thereafter, lobsters were fed following the same method for a further 2 weeks to measure the AD of *Spirulina* protein. Fresh faeces were siphon-collected within the peak time point of egestion (4–8 h post-feeding) onto a 500-μm mesh screen every 0.5 h to minimize the possible leaching. Collected faeces were washed immediately with de-ionized water for 5 s^63^. Rinsed faeces from each lobster were pooled over the 2 weeks, frozen and stored at − 20 °C until chemical analysis.

Apparent digestibility of the reference and test feeds was calculated as:

$$\begin{aligned} {\text{AD}}_{{{\text{DM}}}} \left( \% \right) & = \left( {{1 }{-}{\text{ Y}}_{{{\text{Feed}}}} /{\text{Y}}_{{{\text{Faeces}}}} } \right) \, \times { 1}00 \\ {\text{AD}}_{{{\text{CP}}}} \left( \% \right) & = [{1} - \left( {{\text{X}}_{{{\text{Faeces}}}} /{\text{X}}_{{{\text{Feed}}}} } \right) \, \times \, \left( {{\text{Y}}_{{{\text{Feed}}}} /{\text{Y}}_{{{\text{Faeces}}}} } \right)] \, \times { 1}00 \\ \end{aligned}$$

where AD_DM_ represents the AD of DM in the feed; Y_Feed_ and Y_Faeces_ signify the proportion of the marker (%Y_2_O_3_) in the feed and faeces, respectively; AD_CP_ represents the AD of CP in the feed; X_Feed_ and X_Faeces_ signify the proportion (%) of CP in the feed and faeces, respectively^64^.

Apparent digestibility of CP in the test ingredient *Spirulina* (ADI_CP_) was calculated as:

$${\text{ADI}}_{{{\text{CP}}}} \left( \% \right) = {\text{AD}}_{{{\text{CP}} - {\text{TD}}}} + \left[ {\left( {{\text{AD}}_{{{\text{CP}} - {\text{TD}}}} {-}{\text{AD}}_{{{\text{CP}}}} -_{{{\text{RD}}}} } \right) \times \left( {0.{7} \times {\text{X}}_{{{\text{RD}}}} } \right)/\left( {0.{3} \times {\text{X}}_{{{\text{IN}}}} } \right)} \right]$$

where AD_CP-TD_ and AD_CP-RD_ represent the AD of CP in the test and reference feeds, respectively; X_RD_ and X_IN_ signify the proportion (%) of CP in the reference feed and the test ingredient, respectively^64^.

### Experimental lobsters

After the AD determination, the remaining lobsters in Cage A, B and C, corresponding to CP_40_, CP_50_ and CP_60_ treatments, respectively, were fed ad libitum isoenergetic feeds containing 1% normal *Spirulina* as described above at 08:00 daily for a week. Thereafter, one lobster from each cage was randomly chosen and fasted for three days, then weighed and anaesthetized by immersion in 0 °C seawater^65^. These three lobsters (BW, 1044 ± 121 g, range 812–1220 g, male : female sex ratio = 2:1) were subsequently stored at  − 20 °C for whole-body chemical analysis as described below.

After the 1-week acclimation, 6 lobsters from each of the three treatments (BW, 985 ± 30 g, range 793–1150 g, N = 18) were evenly and randomly chosen for energy metabolism experiments including WBPS determination. The BW among treatments was not statistically different and the sex ratio within treatments was 1:1. Each time two lobsters were weighed and transferred individually into two identical 30 L polyethylene tanks. The experimental conditions were the same as that of the AD determination experiment, and the seawater was aerated by an air-stone connected to the central air supply. The lobsters were fasted for three days to ensure they were at the same post-absorptive status^28^. Before transfer, seawater levels of 16 and 20 L were marked inside of the tank using a marker pen. Thereafter, the seawater level was dropped to 16 L and a lobster (body volume, BV, L) was transferred. The seawater level was again marked to account for 16 L + BV. Then, 4 L of freshly filtered seawater was added and a seawater level of 20 L + BV was marked. The seawater level was kept at 16 L + BV during the fasting period and 20 L + BV during the feeding period, during which the lobster was fed a ^15^N-labeled experimental feed at 1.5% BW. Lobsters that did not consume the entire feed within 30 min were excluded for further analysis.

### Oxygen consumption rate and SDA

During the last 2 h during the 3-day fasting period, the routine metabolic rate (RMR, μmol g^−1^ h^−1^) in each lobster was determined (started from 07:00) based on the measurement of *M*O_2_ (μmol g^−1^ h^−1^)^13,27,42^. Experimental tanks were equipped with a submersible aquarium pump (101 Maxi Pump Power Head 400 L h^−1^, Aqua One, Wallington, Australia) to ensure seawater was well-mixed. Aeration and seawater flow were manually halted for 20 min (*M*O_2_ measurement period), then restarted for 10 min (re-oxygenation period), allowing one *M*O_2_ value recorded per 30 min. Oxygen contents never fell below 70% saturation. A blue transparent solar pool cover (Intex Development Co., Ltd., Hong Kong) was floated on the surface of the seawater during the measuring period to avoid air-seawater gas exchange^31^. During re-oxygenation, the pool cover was removed, and aeration and seawater flow restarted. The halt-restart process was repeated three times and the RMR was determined as the mean of the three *M*O_2_ measurements, where the background *M*O_2_ was subtracted^13,26,27^. Background *M*O_2_ was determined in each tank by the same process described above for 2 h before the lobster was stocked^59,65^.

After consuming all the feed, the seawater level was dropped to 16 L + BV by siphoning 4 L of seawater into a 5-L plastic flask via Tygon E-3603 tubing (Saint-Gobain Performance Plastics, Charny, France). Thereafter, the lobster was subjected to the 20-min halt and 10-min restart cycles at 2 h intervals for the first 12 h, thereafter every 12 h up to 48 h and at 72 h to provide 11 post-prandial *M*O_2_ measurements to examine SDA. Six variables were investigated: (i) peak SDA (SDA_peak_, μmol g^−1^ h^−1^); (ii) time to SDA_peak_ (h); (iii) time when the post-prandial *M*O_2_ returns to RMR (SDA duration, h), determined as two or three consecutive post-prandial *M*O_2_ falling within 1 RMR ± 1 SE; (iv) SDA magnitude (T*M*O_2_, μmol O_2_ g^−1^), calculated by total post-prandial rise of *M*O_2_ above the RMR (T*M*O_2_)^42^; (v) E_SDA_ (energetic cost of SDA, J g^−1^), where SDA magnitude was converted to energy. The E_SDA_ was investigated using three indirect calorimetric approaches^17^. The first two are simplified traditional approaches that multiply measured T*M*O_2_ (mg g^−1^) and an empirical oxycalorific coefficient (Q_ox_) of 13.84 J mg^−1^ O_2_, or a composite Q_ox_^9,66^. The third is the stoichiometric bioenergetic approach, where E_SDA_ (J g^−1^) = 11 × T*M*O_2_ + 2.6 × T*M*CO_2_ − 9.5 × T*M*NH_3_ − 2.44 × T*M*urea, where T*M*CO_2_ (mg g^−1^), T*M*NH_3_ (mg g^−1^) and T*M*urea (mg g^−1^) represent the accumulated CO_2_, NH_3_ and urea excretion during SDA, respectively^9^. (vi) SDA coefficient (C_SDA_, %), calculated by dividing E_SDA_ by the energy in the ingested feed (J g^−1^)^26^.

### Seawater sampling

During the RMR determination, a 20-mL seawater sample was collected within 3 s at the start and end of each measuring period via a 20 mL syringe (Terumo Co., Ltd., Japan) to determine routine excretion rates of total dissolved inorganic carbon (*M*DIC, μmol g^−1^ h^−1^), total ammonia-N (*M*TAN, μmol g^−1^ h^−1^) and urea-N (*M*urea-N, nmol g^−1^ h^−1^), after correction for background levels. Full details of 20-mL seawater sampling were given in Wang et al*.*^9^.

After feeding, the siphon-collected 4-L seawater sample was immediately acidified with 10 mL of 4 M HCl and transferred and stored at 4 °C in a 5-L round bottom glass flask (Schott Duran, Mainz, Germany) for the determination of the initial ^15^N concentration^23,24^. A 20-mL seawater sample was collected at the start and end of each measuring period to determine the post-prandial *M*DIC, *M*TAN and *M*urea-N. At 12, 24, 48 and 72 h post-feeding, faeces were pipetted onto a 500-μm mesh screen following the 20-mL seawater sampling. Pipetting was completed within 1 min. The mesh screen was set on top of the tank to avoid seawater loss from the tank during faecal collection. Subsequently, 4 L of seawater was collected, acidified and stored for the later determination of ^15^N enrichment (expressed as atom percent excess, APE) of ammonia^25,55^. Then 4 L of freshly filtered seawater was added into the tank. The reduction of 20 mL after each sampling was taken into account in post-prandial parameter calculation. After adding fresh seawater for the last time at 72 h post-feeding, the lobster was taken out and weighed. The *M*O_2_, *M*DIC, *M*TAN and *M*urea-N in the tank were measured for 20 min to identify if there were differences in background parameters before and after each experiment^59,65^, and the results showed no differences.

### Instantaneous metabolic energy substrate use calculation

The calculation of instantaneous metabolic energy substrate use has been detailed in Wang et al*.*^9^. Briefly, the fraction of aerobic energy substrate use supplied by protein (amino acid) (P), lipid (L) and carbohydrate (C) was calculated as:

1 $${\text{P }} = {\text{ NQ}}/0.{27}$$

2 $${\text{P }} + {\text{ L }} + {\text{ C }} = { 1}.0$$

3 $${\text{RQ}} = \left( {{\text{m}} - 0.{71}} \right) \times {\text{NQ}}/0.{27} + 0.{29} \times {\text{C}} + 0.{71}$$

where 0.27 is the theoretical maximum nitrogen quotient (NQ) when protein (amino acid) is the only substrate being completely oxidized under aerobic conditions; m is the aerobic respiratory quotient (RQ) for protein (amino acid) oxidation, determined by 0.96 × TAN% + 0.83 × urea-N%, where 0.96 and 0.83 are the aerobic RQ for protein (amino acid) oxidation when ammonia and urea are the unique nitrogenous end-products, respectively; TAN% and urea-N% represent the contribution of *M*TAN and *M*urea-N to *M*TN (total nitrogenous excretion), respectively. The NQ was calculated as *M*TN/*M*O_2_, and the RQ calculated as *M*DIC/*M*O_2_, where *M*TN, *M*O_2_ and *M*DIC were expressed as μmol g^−1^ h^−1^.

### Whole-body protein synthesis

Ammonia from the 4 L collected seawater was distilled into boric acid to form ammonium borate. Full details of ammonia distillation were given in Carter et al*.*^25^ with modification. Briefly, the acidified sample was distilled with 20 anti-bump granules and 80 mL mixture of 8 M NaOH and 0.1 M EDTA. Following distillation, ammonia was trapped as ammonium borate into 10 mL of 1 M boric acid. The ammonium borate was stored at − 20 °C and lyophilized (freeze-dried, FD, − 37 °C) using a freeze dryer (FDA5508, Ilshin Lab Co., Ltd., Korea) to constant weight. The FD ammonium borate samples were used to determine ^15^N enrichment of ammonia to calculate whole-body protein synthesis (WBPS, mg g^−1^ day^−1^), using the endpoint stochastic model^24,25^. Following the WBPS determination, the whole-body fractional protein synthesis rate (k_s_, % day^−1^) was determined using the whole-body protein content data as described by Fraser et al*.*^23^.

### Analysis of chemical composition in feeds, Spirulina, faeces, and whole-body lobster

Feeds, test ingredient *Spirulina*, and faecal samples were FD to constant weight before analysis. Whole-body lobsters were homogenized using a silent cutter (MSK 760-II, Mado, Germany) and three subsamples from each lobster were FD to constant weight. Each FD sample was then finely ground to homogenous powders using a mortar and pestle. For feed and whole-body lobster samples, the content of dry matter (DM), ash, crude protein, total lipid, carbohydrate, and gross energy were determined. For *Spirulina* and faecal samples, DM and crude protein were determined. The DM content of the FD samples was measured gravimetrically following drying at 105 °C for 24 h^67^. Ash content was determined after incineration at 600 °C for 2 h with a combustion oven^67^. All remaining chemical composition analysis including the proportion of Y_2_O_3_ was performed based on the FD samples and corrected for DM^68^.

### Analysis of ^15^N enrichment

The ^15^N enrichment in the experimental feeds and ammonium borate samples was measured by running samples of known nitrogen enrichment alongside the experimental samples using flash combustion isotope ratio mass spectrometry (FCIRMS) (Vario PYRO cube coupled to Isoprime100 mass spectrometer, Elementar Analysensysteme GmbH, Hanau, Germany) at the Central Science Laboratory, University of Tasmania. The ^15^N enrichment in the experimental feeds in CP_40_, CP_50_ and CP_60_ treatments was 1.3, 1.1 and 0.89 APE, respectively.

### Data analysis

All figures were plotted and all statistical analysis performed using GraphPad Prism V6.0 (GraphPad Software Inc., San Diego, CA, USA). A probability of *P* < 0.05 was considered significant in all analyses. Before statistical analyses normality tests were carried out via Kolmogorov–Smirnov test, followed by the verification of homogeneity of variances via Bartlett’s test. Homogeneous data were compared using t-tests and one-way analysis of variance (ANOVA), heterogeneous data were compared using the Kruskal–Wallis test. The relationship between the cumulative rate of ^15^N-labeled ammonia excretion after feeding (ce*, %, expressed as the percentage of ^15^N in the assimilated feed) and time (t) was estimated using a nonlinear regression model and described by ce* = a(1 − e^−bt^), where a and b are constants^24,25^. Differences in ce* over time in each treatment were examined using a one-way repeated-measures ANOVA to identify the period of constant ^15^N-labeled ammonia excretion, followed by post-hoc Tukey HSD test to enable the WBPS calculation to be made with the least overestimation^23,24^. All data were expressed as mean ± SE, except that the chemical composition, the AD of *Spirulina* protein, and the fractional data of instantaneous metabolic energy substrate use were presented as mean values. The first step to calculate metabolic energy substrate use was to filter the outliers of *M*O_2_, *M*DIC, *M*TAN and *M*urea-N, which might cause the RQ and/or NQ above the theoretical maximums under aerobic conditions^15,69^, likely due to spontaneous activity^70,71^. Outliers were filtered using the interquartile range (IQR), the most commonly used outlier-resistant method in aquatic ectotherms^72–74^.

## Supplementary Information

Supplementary Information.

## Data Availability

The datasets generated during this study are available in the Supplementary Information files.
